# Safety of Lowering the Platelet Transfusion Threshold Before Central Venous Catheterisation in Patients With Haematological Diseases

**DOI:** 10.1111/ejh.70287

**Published:** 2026-08-07

**Authors:** Mathias Lazarevic Lindblad, Elin Pålsson, Vladimir Lazarevic, Leila Naddi, Ola Borgquist, Lars Nilsson, Maria Adrian, Björn P. Bark, Thomas Kander

**Affiliations:** ^1^ Department of Clinical Sciences Lund University Lund Sweden; ^2^ Department of Intensive and Perioperative Care Skåne University Hospital Lund Sweden; ^3^ Department of Haematology, Oncology and Radiation Physics Skåne University Hospital Lund Sweden; ^4^ Department of Cardiothoracic Surgery Anaesthesia and Intensive Care Skåne University Hospital Lund Sweden; ^5^ Department of Anaesthesia and Perfusion The Prince Charles Hospital Brisbane Queensland Australia

**Keywords:** bleeding, central venous catheterisation, haematological diseases, platelet transfusion, thrombocytopenia, vascular access

## Abstract

**Introduction:**

Central venous catheter (CVC) insertion is frequently required in patients with haematological diseases. Despite limited evidence, thrombocytopenia often prompts prophylactic platelet transfusion before catheterisation.

**Methods:**

We conducted an observational before‐and‐after study including the first non‐tunnelled CVC insertion performed in adult patients with haematological diseases between 2013 and 2026 at the Department of Haematology, Skåne University Hospital, Lund, Sweden. In March 2023, the recommended platelet transfusion threshold before CVC insertion was lowered from < 50 × 10^9^/L to < 10 × 10^9^/L. The primary outcome was the proportion of grade 3–4 bleeding events. Secondary outcomes included the proportion of grade 2 bleeding events and transfusion practices.

**Results:**

A total of 992 patients were included (715 before and 277 after implementation). Grade 3–4 bleeding events occurred in 14/715 procedures before implementation and in none of the 277 insertions after implementation (absolute risk difference −2.0%, 95% CI: −3.3 to −0.4; *p* = 0.014). Grade 2 bleeding events were unchanged (8.5% vs. 6.5%, absolute risk difference −2.0%, 95% CI: −5.2 to 2.0; *p* = 0.289). Preprocedural platelet transfusions decreased substantially (30% vs. 6.5%; *p* < 0.001).

**Conclusions:**

Lowering the platelet transfusion threshold to < 10 × 10^9^/L was not associated with increased bleeding complications and reduced platelet transfusion use. These findings support a more restrictive preprocedural transfusion strategy.

## 1 Introduction

Patients with haematological diseases often need central venous catheters (CVCs) for essential treatments, but their underlying haemostatic abnormalities may increase the risk of bleeding in association with catheterisation [1, 2]. Despite this presumed risk, the actual incidence of clinically significant bleeding following CVC insertion in patients with haematological diseases remains uncertain. In the PACER trial, omitting prophylactic platelet transfusion in patients with platelet counts of 10–50 × 10^9^/L undergoing CVC placement increased grade 2–4 bleeding, driven by grade 2 events, but not clinically significant (grade 3–4) bleeding (see Table 1), suggesting that lower platelet thresholds may be sufficient to prevent serious bleeding events [3]. When performed by experienced operators using ultrasound guidance and standardised techniques, CVC insertion has been associated with a low bleeding risk in patients with thrombocytopaenia [4, 5, 6, 7].

**TABLE 1:** Definitions of central venous catheterisation‐related bleeding events according to modified Common Terminology Criteria for Adverse Events classifications.

Bleeding grade	Definition
Grade 0	No bleeding.
Grade 1	Slight oozing from the insertion site not requiring intervention.
Grade 2	Bleeding requiring prolonged external compression.
Grade 3	Bleeding requiring transfusions or subacute invasive measures.
Grade 4	Life threatening condition in need of urgent intervention.

Preprocedural platelet transfusion thresholds vary between 10 and 50 × 10^9^/L across guidelines [8, 9, 10, 11, 12], and adherence to these recommendations varies [13]. As all transfusions carry risks and increase use of resources, the interest in more restrictive strategies is growing. In line with this, Skåne University Hospital, Lund, Sweden, lowered the recommended platelet transfusion threshold before CVC insertion from < 50 to < 10 × 10^9^/L on 1 March 2023, based on the hypothesis that procedural factors, rather than low platelet count alone, are the primary determinants of clinically significant bleeding risk.

This observational study aimed to evaluate the impact of lowering the platelet transfusion threshold from < 50 × 10^9^/L to < 10 × 10^9^/L before CVC insertion. The primary outcome was the proportion of clinically significant (grade 3–4) bleeding events following catheter insertion, compared between the periods before and after implementation of the revised transfusion policy. Secondary analyses evaluated differences in grade 2 bleeding events, platelet transfusion practices, and other bleeding and mechanical complication outcomes.

## 2 Methods

This observational study was approved by the Swedish Ethical Review Authority (reference numbers 2018/295, 2020‐06674, and 2023‐07778‐02). As the study involved the observation of a current clinical practice, the requirement for written informed consent was waived. The manuscript was prepared in accordance with the STROBE guidelines for observational studies [14]. The study was registered at ClinicalTrials.gov (NCT06187831).

All adult patients (≥ 18 years) undergoing a first non‐tunnelled CVC insertion at the Department of Haematology, Skåne University Hospital, Lund, Sweden, between 28 May 2013 and 28 February 2026 were eligible for inclusion. For patients with multiple CVC insertions during the study period, only the first insertion was included. The study start date corresponded to the availability of prospectively recorded CVC data in the electronic health record. Patients were divided into pre‐ and post‐implementation cohorts based on a revised transfusion policy introduced on 1 March 2023, lowering the preprocedural platelet transfusion threshold from < 50 to < 10 × 10^9^/L. Outcomes were compared between periods. CVC insertions were performed according to regional guidelines, as previously described [15, 16]. Prophylactic tranexamic acid was not routinely administered. There were no changes to the institutional red blood cell transfusion policy during the study period.

### 2.1 Data Collection

Raw data were extracted from the electronic health record (Melior, Oracle Health, North Kansas City, MO, USA) using a structured query language script targeting standardised CVC insertion and care templates. Uncertainties were resolved by chart review and consensus within the author group. The same predefined variables and data extraction procedures were used throughout the study period. In 2022, an updated CVC insertion template was implemented in the electronic health record. Variables not available in the structured dataset were manually retrieved and merged into the study database (Microsoft Excel, Version 16, Microsoft, Redmond, WA, USA).

### 2.2 Outcomes and Definitions

Bleeding events were classified according to a modification of the Common Terminology Criteria for Adverse Events (version 6.0) in accordance with similar publications and presented in Table 1 [3, 4, 17].

The primary outcome was the proportion of grade 3–4 bleeding events. Secondary outcomes were proportions of grade 2 bleeding events, preprocedural platelet transfusions, number of preprocedural platelet units, as well as other mechanical complications (pneumothorax, arterial puncture, arrhythmias, and persistent nerve damage).

Although grade 1 bleeding events often occur during CVC insertion, they are clinically insignificant, require no intervention, are seldom documented, and were therefore not included as an outcome in this study.

#### 2.2.1 Monitoring Grade 3 and 4 Bleeding Events

Following implementation of the revised transfusion policy, predefined safety monitoring criteria were applied. The platelet transfusion threshold of < 10 × 10^9^/L was maintained provided that the incidence of post‐procedural grade 3–4 bleeding events in patients with preprocedural platelet counts of 10–49 × 10^9^/L remained below prespecified limits. These limits were defined as ≤ 2 events within 6 months, ≤ 3 within 1 year, or ≤ 4 within 2 years. If these limits were exceeded, the threshold would have been increased to < 30 × 10^9^/L.

### 2.3 Statistics and Analyses

The sample size was determined by the total number of CVC insertions during the study period. As this was an observational implementation study including all eligible patients during the study period, no formal a priori sample size calculation was performed.

Continuous variables with a normal distribution are presented as mean with standard deviation (SD), while skewed variables are presented as median with interquartile range (IQR). Normality was assessed using histograms, comparison of means and medians, and the Shapiro–Wilk test. Between‐group comparisons for continuous variables were performed using independent samples *t*‐test or Mann–Whitney *U* test. Categorical variables are presented as number (proportion) and analysed using Chi‐square or Fisher's exact test as appropriate.

For binary outcomes, absolute risk differences (ARD) with 95% confidence intervals (95% CI) were calculated using the Newcombe score method. As this method does not provide corresponding *p*‐values, *p*‐values were derived separately using Chi‐square tests without continuity correction or Fisher's exact test, as appropriate. For individual proportions when data were sparse (e.g., zero events), exact Clopper–Pearson 95% confidence intervals were reported. For continuous outcomes, Hodges–Lehmann estimates with 95% CI were derived from the Wilcoxon rank‐sum test. Analyses were exploratory and prioritised sensitivity to detect potential signals; no adjustment for multiple testing was performed to avoid increasing type II error risk. All tests were two‐sided, and *p*‐values < 0.05 were considered statistically significant.

Sensitivity analyses (presented in the Tables S1–S4) were performed by restricting the cohort to procedures performed between January 2020 and February 2026 to reduce the impact of temporal changes in procedural practice, and by analysing the predefined subgroup with preprocedural platelet counts of 10–50 × 10^9^/L corresponding to the PACER trial population [3].

Analyses were performed in R (version 4.5.3) using RStudio (version 2026.01.1 + 403, Posit PBC, Boston, MA, USA).

## 3 Results

In total, 992 patients were included, of whom 715 (72%) underwent their first CVC insertion before and 277 (28%) after implementation of the revised platelet transfusion threshold. Baseline characteristics stratified by study period are presented in Table 2.

**TABLE 2:** Patient characteristics and procedural data before and after implementation of the revised platelet transfusion threshold prior to central venous catheter insertion. Values are number (proportion), mean (SD) or median (IQR [range]).

	Overall, *n* = 992	Before new policy, *n* = 715	After new policy, *n* = 277	*p*
Age; years	56 (16)	56 (16)	56 (16)	0.672
BMI; kg/m^2^	26 (4.9)	26 (4.9)	27 (4.9)	0.147
Missing	36 (3.6%)	26 (3.6%)	10 (3.6%)	0.984
Gender; female	428 (43%)	294 (41%)	134 (48%)	0.038
Diagnosis				
AML	383 (39%)	280 (39%)	103 (37%)	0.566
ALL	107 (11%)	77 (11%)	30 (11%)	0.978
CML	28 (2.8%)	21 (2.9%)	7 (2.5%)	0.726
CLL	29 (2.9%)	26 (3.6%)	3 (1.1%)	0.032
Lymphoma	115 (12%)	83 (12%)	32 (12%)	0.980
MDS	50 (5.0%)	30 (4.2%)	20 (7.2%)	0.051
MPN	9 (0.9%)	8 (1.1%)	1 (0.4%)	0.458
Multiple myeloma	150 (15%)	94 (13%)	56 (20%)	0.005
Other^a^	121 (12%)	96 (13%)	25 (9.0%)	0.057
Vein for insertion				
Subclavian/axillary	772 (79%)	552 (78%)	220 (81%)	0.431
Internal jugular	204 (21%)	151 (21%)	53 (19%)	0.489
External jugular	1 (0.1%)	1 (0.1%)	0 (0.0%)	1.000
Femoral	1 (0.1%)	1 (0.1%)	0 (0.0%)	1.000
Missing	14 (1.4%)	10 (1.4%)	4 (1.4%)	1.000
Side of insertion; right	644 (66%)	410 (58%)	234 (86%)	< 0.001
Missing	15 (1.9%)	11 (2.7%)	4 (1.0%)	0.089
Catheter type				
1 lm	414 (43%)	408 (59%)	6 (2.2%)	< 0.001
2 lm	494 (51%)	241 (35%)	253 (93%)	< 0.001
3 lm	11 (1.1%)	4 (0.6%)	7 (2.6%)	0.015
5 lm	4 (0.4%)	4 (0.6%)	0 (0.0%)	0.582
Dialysis	43 (4.5%)	37 (5.3%)	6 (2.2%)	0.034
Missing	26 (2.6%)	21 (2.9%)	5 (1.8%)	0.317
Needle passes				
1	692 (74%)	480 (72%)	212 (78%)	0.041
2	154 (16%)	120 (18%)	34 (13%)	0.043
3	60 (6.4%)	45 (6.7%)	15 (5.5%)	0.498
≥ 4	34 (3.6%)	24 (3.6%)	10 (3.7%)	0.939
Missing	52 (5.2%)	46 (6.4%)	6 (2.2%)	0.007
Insertion method				
Real‐time ultrasound	859 (88%)	588 (84%)	271 (99%)	< 0.001
Other^b^	118 (12%)	116 (16%)	2 (0.7%)	< 0.001
Missing	15 (1.5%)	11 (1.5%)	4 (1.4%)	1.000
Operator experience > 5 years	707 (74%)	503 (73%)	204 (75%)	0.609
Missing	31 (3.1%)	27 (3.8%)	4 (1.4%)	0.058
Treated with anticoagulants^c^	78 (7.9%)	58 (8.1%)	20 (7.2%)	0.640
Prophylactic tranexamic acid	7 (0.7%)	6 (0.8%)	1 (0.4%)	0.680
Haemoglobin, g/L	95 (85–110 [38–150])	96 (86–110 [49–150])	93 (82–110 [38–150])	0.231
Missing	120 (12%)	96 (13%)	24 (8.7%)	0.039
INR	1.1 (1–1.2 [0.8–3.8])	1.1 (1–1.2 [0.8–3.8])	1.1 (1–1.2 [0.8–2.3])	0.108
Missing	157 (16%)	126 (18%)	31 (11%)	0.013
aPTT; s	26 (24–28 [20–66])	26 (24–28 [20–54])	26 (24–28 [20–66])	0.531
Missing	180 (18%)	130 (18%)	50 (18%)	0.962
Fibrinogen, g/L	3.5 (2.6–4.4 [0.3–9])	3.5 (2.6–4.3 [0.3–9])	3.5 (2.4–4.4 [0.3–9])	0.971
Missing	421 (42%)	326 (46%)	95 (34%)	0.001
Preprocedural PLC; × 10^9^/L	82 (34–190 [5–820])	78 (34–190 [5–820])	96 (38–200 [5–730])	0.271
Missing	56 (5.6%)	42 (5.9%)	14 (5.1%)	0.616
Preprocedural PLC < 10 × 10^9^/L	35 (3.7%)	27 (4.0%)	8 (3.0%)	0.482
Preprocedural PLC 10–50 × 10^9^/L	297 (32%)	215 (32%)	82 (31%)	0.821
Preprocedural PLC > 50 × 10^9^/L	604 (65%)	431 (64%)	173 (66%)	0.617
aPTT > 43 s	12 (1.5%)	6 (1.0%)	6 (2.6%)	0.105
INR > 1.8	10 (1.2%)	6 (1.0%)	4 (1.6%)	0.492

Abbreviations: ALL, acute lymphoblastic leukaemia; AML, acute myeloid leukaemia; aPTT, activated partial thromboplastin time; CLL, chronic lymphocytic leukaemia; CML, chronic myeloid leukaemia; INR, international normalised ratio; MDS, myelodysplastic syndrome; MPN, myeloproliferative neoplasm; PLC, platelet count.

^a^ Seminoma and non‐seminoma, sickle cell anaemia, Erdheim–Chester disease, aplastic anaemia, multiple sclerosis, hemophagocytic lymphohistiocytosis, myelofibrosis, mixed‐phenotype acute leukaemia, acquired haemophilia, and plasmacytoma.

^b^ Change over wire, anatomic landmark technique, and ultrasound guidance only before insertion.

^c^ Platelet inhibitors, warfarin, non‐vitamin K antagonist oral anticoagulants within 3 days before CVC insertion, or low‐molecular‐weight heparin (therapeutic doses within 24 h or prophylactic doses within 12 h before the procedure).

Patient characteristics were broadly comparable between groups. The distribution of haematological diagnoses was generally consistent, with acute myeloid leukaemia being the most common diagnosis (39%).

Procedural characteristics differed between periods. The subclavian/axillary vein was the most frequently used insertion site in both periods (78% before vs. 81% after). However, right‐sided insertions were more common after implementation (58% before vs. 86% after; *p* < 0.001). Real‐time ultrasound guidance was also more common after implementation (84% before vs. 99% after; *p* < 0.001). Catheter type distribution differed, with a shift from one‐lumen (59% before vs. 2.2% after; *p* < 0.001) to two‐lumen catheters (35% before vs. 93% after; *p* < 0.001). Dialysis catheters were more common before implementation (5.3% before vs. 2.2% after; *p* = 0.034), whereas operator experience was similar between periods (73% before vs. 75% after). The median preprocedural platelet count was similar between periods (78 (34–190 [5–820]) vs. 96 (38–200 [5–730]) × 10^9^/L; *p* = 0.271), as was the distribution across clinically relevant platelet count thresholds (< 10, 10–50 and > 50 × 10^9^/L).

Outcomes are presented in detail in Table 3. In summary, 14 grade 3–4 bleeding events occurred before implementation (2.0%; 95% CI: 1.1–3.3), whereas no grade 3–4 bleeding events occurred after implementation (0.0%; 95% CI: 0.0–1.3), corresponding to an ARD of −2.0% (95% CI: −3.3 to −0.4; *p* = 0.014). Of the 11 grade 3 bleeding events, three were classified as grade 3 because they required a subacute invasive intervention, whereas eight were classified as grade 3 because they required red blood cell transfusion. The three grade 4 bleeding events required urgent intervention and observation in the intensive care unit. The proportion of grade 2 bleeding events was similar between groups (8.5% vs. 6.5%, ARD −2.0%, 95% CI: −5.3 to 2.0; *p* = 0.289). Given the absence of grade 3–4 bleeding events in the post‐implementation group, multivariable adjustment was not feasible because of complete separation; analyses of the primary outcome were therefore limited to unadjusted comparisons.

**TABLE 3:** Bleeding, transfusion and mechanical complication outcomes before and after implementation of the revised platelet transfusion threshold. Values are number (proportion), number/total (proportion) or median (IQR [range]).

	Overall *n* = 992	Before new policy, *n* = 715	After new policy, *n* = 277	ARD or median difference	95% CI	*p*
Primary outcomes						
Grade 3 bleeding event; *n* (% (95% CI))^a^	11 (1.1% (0.6–2.0))	11 (1.5% (0.8–2.7))	0 (0.0% (0.0–1.3))	−1.5	−2.7 to −0.0	0.041
Grade 4 bleeding event; *n* (% (95% CI))^a^	3 (0.3% (0.1–0.9))	3 (0.4% (0.1–1.2))	0 (0.0% (0.0–1.3))	−0.4	−1.2 to 1.0	0.564
Grade 3–4 bleeding event; *n* (% (95% CI))^a^	14 (1.4% (0.8–2.4))	14 (2.0% (1.1–3.3))	0 (0.0% (0.0–1.3))	−2.0	−3.3 to −0.4	0.014
Secondary outcomes						
Grade 2 bleeding event	79 (8.0%)	61 (8.5%)	18 (6.5%)	−2.0	−5.3 to 2.0	0.289
Preprocedural PLC (transfused insertions); × 10^9^/L^b^	22 (15–39 [5–110])	23 (15–40 [5–110])	16 (10–21 [5–56])	8.0	1.0 to 15.0	0.015
CVC insertions within each PLC interval receiving preprocedural platelet transfusion						
All	231 (23%)	213 (30%)	18 (6.5%)	−23.3	−27.5 to −18.5	< 0.001
< 10 × 10^9^/L	30/35 (86%)	25/27 (93%)	5/8 (62%)	−30.1	−62.5 to −1.4	0.067
10–50 × 10^9^/L	178/297 (60%)	166/215 (77%)	12/82 (15%)	−62.6	−70.5 to −51.5	< 0.001
> 50 × 10^9^/L	23/604 (3.8%)	22/431 (5.1%)	1/173 (0.6%)	−4.5	−7.1 to −1.4	0.008
Platelet transfusion within 24 h after CVC	129 (13%)	95 (13%)	34 (12%)	−1.0	−5.3 to 3.9	0.671
Preprocedural units of platelet transfusions						
No transfusion	761 (77%)	502 (70%)	259 (94%)	23.3	18.5 to 27.5	< 0.001
One unit	211 (21%)	196 (27%)	15 (5.4%)	−22.0	−26.0 to −17.4	< 0.001
Two units	20 (2.0%)	17 (2.4%)	3 (1.1%)	−1.3	−2.9 to 0.9	0.312
Total units	251	230	21	n/a	n/a	n/a
Per patient	0.25	0.32	0.08	n/a	n/a	n/a
Other mechanical complications						
Pneumothorax	6 (0.6%)	4 (0.6%)	2 (0.7%)	0.2	−0.9 to 2.1	0.674
Arterial puncture	20 (2.0%)	19 (2.7%)	1 (0.4%)	−2.3	−3.8 to −0.4	0.021
Non‐persistent nerve injury	3 (0.3%)	2 (0.3%)	1 (0.4%)	0.1	−0.7 to 1.7	1.000
Arrhythmias requiring intervention	0 (0.0%)	0 (0.0%)	0 (0.0%)	0.0	−0.5 to 1.4	1.000

Abbreviations: ARD, absolute risk difference; CVC, central venous catheter; n/a, not applicable; PLC, platelet count.

^a^ Clopper–Pearson 95% CI are presented for proportions with sparse data or zero events.

^b^ Median difference presented as Hodges–Lehmann estimate with 95% CI.

Following implementation of the revised transfusion policy, the use of preprocedural platelet transfusions decreased substantially, both in overall proportion (30% vs. 6.5%; *p* < 0.001) and in the number of administered platelet units. Among transfused patients, the median preprocedural platelet count was lower after implementation (23 vs. 16 × 10^9^/L; *p* = 0.015). There was no difference between groups in postprocedural platelet transfusions within 24 h of catheterisation. The mean number of preprocedural platelet units transfused decreased from 0.32 to 0.08 platelet units per patient, corresponding to an approximate cost reduction of £95 per CVC insertion (unit cost £400 in March 2026).

Mechanical complications were rare and largely comparable between groups. However, arterial puncture occurred less frequently after implementation (2.7% vs. 0.4%; ARD −2.3%, 95% CI: −3.8 to −0.4; *p* = 0.021).

Clinical and procedural characteristics of patients with grade 3–4 bleeding events are presented in Table S7 together with those of patients without grade 2–4 bleeding events. Patients with grade 3–4 bleeding had a median preprocedural platelet count of 27 (16–49 [11–85]) × 10^9^/L, and 10 of 14 (71%) received a preprocedural platelet transfusion.

### 3.1 Sensitivity Analyses

Sensitivity analyses restricted to procedures performed between January 2020 and February 2026, during which ultrasound guidance was used in 99% of insertions in both groups, showed no increase in bleeding after implementation of the revised transfusion policy (Tables S1 and S2). The number of grade 3–4 bleeding events was lower than in the overall cohort, and the difference between periods was not statistically significant. Platelet transfusions within 24 h after CVC insertion were, however, more frequent post‐implementation (7.1% vs. 12%; *p* = 0.029). Among patients receiving postprocedural platelet transfusions, most had preprocedural platelet counts of 10–50 × 10^9^/L in both periods (62% before vs. 76% after) (Table S4).

In a sensitivity analysis restricted to patients with preprocedural platelet counts of 10–50 × 10^9^/L, the proportion of grade 2 bleeding events did not differ between periods (13% vs. 9.8%, ARD −3.3%, 95% CI: −10.3 to 5.9; *p* = 0.441). Grade 3–4 bleeding events occurred in 4.7% of patients before implementation and in none after (ARD −4.7%, 95% CI: −8.3 to 0.3; *p* = 0.067) (Table S3). Analyses including all catheter insertions yielded results consistent with the primary analysis, with no increase in bleeding after implementation and a substantial reduction in preprocedural platelet transfusions (Tables S5 and S6).

## 4 Discussion

In this observational study of 992 patients with haematological diseases, we evaluated the impact of lowering the platelet transfusion threshold from < 50 to < 10 × 10^9^/L before CVC insertion. The revised transfusion policy was not associated with an increased incidence of grade 3–4 or grade 2 bleeding events. A marked reduction in preprocedural platelet transfusions overall, particularly among patients with preprocedural platelet counts of 10–50 × 10^9^/L, indicated good adherence to the revised transfusion policy.

Our findings should be interpreted in the context of the PACER trial, which reported a higher incidence of grade 2–4 bleeding without platelet transfusion in patients with preprocedural platelet counts of 10–50 × 10^9^/L [3]. However, interpretation of the PACER trial results is limited by potential bias. Although grade 3 bleeding was defined by the need for red blood cell transfusion and/or procedural intervention, no cases required intervention. This means that all grade 3 events were classified as such based on red blood cell transfusion alone in already anaemic patients, which in turn raises questions about clinical relevance. In addition, transfusion decisions were likely made by clinicians aware of patients' transfusion status, as blinding was not reported, introducing a risk of bias in outcome assessment. Importantly, no difference in grade 3 bleeding events was observed, and the findings were primarily driven by grade 2 events. In contrast, this study found no increase in either grade 2 or grade 3–4 bleeding after lowering the transfusion threshold. Notably, in a sensitivity analysis restricted to patients with preprocedural platelet counts of 10–50 × 10^9^/L, corresponding to the PACER population, no increase in grade 2 bleeding was observed and no grade 3–4 bleeding events occurred after implementation. Although event numbers were small, they were consistent with the limited number of events reported in the PACER trial. Together, these findings suggest that a more restrictive transfusion approach appears safe in routine clinical practice.

In the current study, no grade 3–4 bleeding events were observed following the revised policy implementation, and the proportion of grade 2 bleeding events remained unchanged despite significantly lower preprocedural platelet count. One possible explanation is the increased use of real‐time ultrasound guidance over time, which has been shown to reduce mechanical complications including bleeding [8, 18]. However, sensitivity analyses restricted to later years (2020–2026), during which the use of ultrasound guidance for CVC insertion was the same between groups, yielded similar results, with no increase in bleeding risk despite lower platelet thresholds. These findings suggest that the observed safety cannot be explained solely by improvements in procedural technique, nor by operator experience, as no differences were observed in operator experience between the periods. However, operator experience, defined as > 5 vs. ≤ 5 years, may not fully capture procedural expertise, particularly as more experienced operators may not have been trained in real‐time ultrasound‐guided infraclavicular CVC insertion. In addition, procedural standardisation at the study centre has evolved over time and is not fully captured by the variables included in this analysis. As shown by previous data from the same centre, the use of ultrasound guidance has increased over time. In Bjorkander et al. ultrasound use during CVC insertion in the same region of Sweden was 49%, compared with 93% in the later (2022) CVC‐MECH study performed in the same cohort [15, 19]. The latter was conducted after implementation of an updated CVC insertion template in the electronic health record, which improved adherence to documentation [20]. In the present study, ultrasound guidance was used in 99% of procedures in the post‐implementation period, consistent with a gradual adoption of the technique at the study centre. Increasing familiarity with a standardised approach to subclavian/axillary catheterisation may have contributed to improved procedural safety.

Although the overall number of bleeding events was low, it was higher in the whole cohort of the present study than that reported in the general population from the same region of Sweden. Adrian et al. reported 419 (3.3%) grade 2 events and 9 (0.07%) grade 3–4 events among 12 667 CVC insertions, compared with 79 (8.0%) grade 2 events and 14 (1.4%) grade 3–4 events in the present study [15]. This suggests that underlying impaired blood clotting in patients with haematological diseases probably continues to influence bleeding risk, despite improvements in procedural technique.

Unexpectedly, the proportion of grade 2 bleeding events did not increase following implementation of the new policy, despite a shift towards the use of larger‐diameter catheters. In December 2020, the standard CVC for patients with haematological diseases at our institution changed from single‐ to double‐lumen catheters [21]. Single‐lumen catheters typically do not require tract dilation or skin incision, whereas insertion of double‐lumen catheters involves tract dilation and may require a small incision to facilitate placement, which would be expected to increase the risk of minor bleeding complications. The absence of such an effect further supports the notion that procedural factors, rather than platelet count alone, are key determinants of clinically relevant bleeding risk.

The proportion of platelet transfusions within 24 h after CVC insertion was similar between groups (13% before vs. 12% after), and comparable to that reported in the PACER trial [3]. In a sensitivity analysis restricted to procedures performed between 2020 and 2026 (Table S2), however, the proportion was higher after implementation (7.1% before vs. 12% after; *p* = 0.029). Most patients receiving postprocedural platelet transfusions had preprocedural platelet counts of 10–50 × 10^9^/L in both periods (Table S4). Importantly, this was not accompanied by an increase in grade 3–4 or grade 2 bleeding events.

Platelet transfusions are associated with a relatively high rate of transfusion reactions and are among the most frequently implicated components [22]. Reduced exposure to platelet transfusions lowers the risk of transfusion‐related complications, which could further decrease healthcare costs and improve clinical outcomes [23]. In addition, limiting platelet exposure may reduce the risk of platelet refractoriness, a clinically important complication in patients with haematological diseases who frequently require repeated platelet transfusions [24]. The revised platelet transfusion threshold reduced the mean number of platelet units per patient from 0.32 to 0.08, potentially saving £95 per catheter insertion (March 2026).

Despite these findings, the optimal platelet transfusion threshold prior to low‐bleeding‐risk invasive procedures remains uncertain. The ongoing Thresholds for Platelets (T4P) trial (ISRCTN79371664), a large multicentre randomised study evaluating different transfusion thresholds for invasive procedures, including CVC insertions, is expected to further inform platelet transfusion strategies [25]. However, this study primarily includes critically ill patients rather than patients with haematological diseases.

### 4.1 Strengths and Limitations

This study has several strengths. The large, real‐world cohort of 992 patients with haematological diseases provides a robust and comprehensive dataset, an uncommon occurrence in this patient group. In addition, the combination of detailed electronic health record extraction and manual verification ensured high data accuracy.

A key limitation is the before‐and‐after design, which limits the ability to fully account for time‐dependent confounders and temporal changes in procedural practice, despite the sensitivity analyses performed. Furthermore, this was a retrospective study of performed CVC insertions. Procedures that were deferred, including those postponed because of ongoing thrombocytopenia‐related bleeding, were not captured and therefore could not be quantified. Consequently, our findings may not be generalisable to patients with active bleeding at the time CVC insertion is considered. The sample size was determined by the number of eligible patients during the study period; consequently, the low incidence of clinically significant bleeding events may have limited the statistical power to detect clinically relevant differences between groups, increasing the risk of type II error. To avoid masking potential differences between the pre‐ and post‐implementation cohorts, no adjustment for multiple testing was applied. While this approach facilitates the identification of potentially meaningful differences in baseline characteristics, it may increase the risk of type I error. In addition, post‐transfusion platelet counts were not consistently measured before CVC insertion, leaving the true platelet count at the time of the procedure unknown in transfused patients. Finally, the generalisability of the findings to centres with greater variability in operator experience and catheterisation techniques may be limited.

## 5 Conclusion

Lowering the platelet transfusion threshold from < 50 to < 10 × 10^9^/L did not increase the number of bleeding complications related to CVC insertion in patients with haematological diseases. The findings support the use of a more restrictive transfusion strategy and the hypothesis that procedural factors, rather than low preprocedural platelet count alone, may be the predominant determinants of clinically significant bleeding risk.

## Author Contributions

Conceptualization and methodology: M.L.L., O.B., T.K.; investigation: M.L.L., E.P.; data curation, formal analysis, project administration, and original draft preparation: M.L.L.; supervision: V.L., L.N., M.A., B.P.B., T.K.; review and editing: all authors. All authors approved the final manuscript.

## Funding

This study was supported by the Swedish Government Grant for Clinical Research Within the Swedish National Health Service (ALF Grant number 748404) (T.K.), a Regional Research Grant from Region Skåne, Sweden (T.K.), LÖF (Landstingens Ömsesidiga Försäkringsbolag), Sweden (TK), the Swedish Society of Medicine (TK), and a Regional Research Grant from Region Skåne, Sweden (L.N.).

## Ethics Statement

The study was approved by the Swedish Ethical Review Authority (reference numbers 2018/295, 2020‐06674, and 2023‐07778‐02).

## Consent

As this observational study involved evaluation of current clinical practice, the requirement for written informed consent was waived by the Swedish Ethical Review Authority.

## Conflicts of Interest

The authors declare no conflicts of interest.

## Supporting information


**Table S1:** Patient characteristics and procedural data before and after implementation of the revised platelet transfusion threshold in procedures performed between 2020 and 2026. Values are number (proportion), mean (SD) or median (IQR [range]).
**Table S2:** Bleeding, transfusion and mechanical complication outcomes before and after implementation of the revised platelet transfusion threshold in procedures performed between 2020 and 2026. Values are number (proportion), number/total (proportion) or median (IQR [range]).
**Table S3:** Bleeding events before and after implementation of the revised platelet transfusion threshold in central venous catheter insertions with preprocedural platelet counts of 10–50 × 10^9^/L. Values are number (proportion).
**Table S4:** Platelet transfusions administered within 24 h after central venous catheter insertion according to preprocedural platelet count before and after implementation of the revised platelet transfusion threshold in procedures performed between 2020 and 2026. Values are number (proportion) or number/total (proportion).
**Table S5:** Patient characteristics and procedural data before and after implementation of the revised platelet transfusion threshold, including all catheter insertions. Values are number (proportion), mean (SD) or median (IQR [range]).
**Table S6:** Bleeding, transfusion and mechanical complication outcomes before and after implementation of the revised platelet transfusion threshold, including all catheter insertions. Values are number (proportion), number/total (proportion) or median (IQR [range]).
**Table S7:** Patient characteristics and procedural data for patients with grade 3–4 bleeding events. Values are number (proportion), mean (SD) or median (IQR [range]).

## Data Availability

The data and statistical code that support the findings of this study are available from the corresponding author upon reasonable request.
