# Supplementary material for: Safety of Lowering the Platelet Transfusion Threshold Before Central Venous Catheterisation in Patients With Haematological Diseases
Source: Eur J Haematol. 2026 Aug 7;117(6):1531–40. doi: 10.1111/ejh.70287 (PMC13729923; doi:10.1111/ejh.70287)
Supplement: Supplementary file 1 [file EJH-117-1531-s001.docx]

**Supplementary Tables S1–S7**

**Table of contents**

**Table S1 – Patient characteristics and procedural data before and after implementation of the revised platelet transfusion threshold in procedures performed between 2020 and 2026. Values are number (proportion), mean (SD) or median (IQR [range]).**

**Table S2 – Bleeding, transfusion and mechanical complication outcomes before and after implementation of the revised platelet transfusion threshold in procedures performed between 2020 and 2026.** **Values are number (proportion), number/total (proportion) or median (IQR [range]).**

**Table S3 – Bleeding events before and after implementation of the revised platelet transfusion threshold in central venous catheter insertions with preprocedural platelet counts of 10–50 × 10⁹/l.** **Values are number (proportion).**

**Table S4 – Platelet transfusions administered within 24 h after central venous catheter insertion according to preprocedural platelet count before and after implementation of the revised platelet transfusion threshold in procedures performed between 2020 and 2026.** **Values are number (proportion) or number/total (proportion).**

**Table S5 – Patient characteristics and procedural data before and after implementation of the revised platelet transfusion threshold, including all catheter insertions.** **Values are number (proportion), mean (SD) or median (IQR [range]).**

**Table S6 – Bleeding, transfusion and mechanical complication outcomes before and after implementation of the revised platelet transfusion threshold, including all catheter insertions.** **Values are number (proportion), number/total (proportion) or median (IQR [range]).**

**Table S7 – Patient characteristics and procedural data for patients with grade 3–4 bleeding events.** **Values are number (proportion), mean (SD) or median (IQR [range]).**

**Table S1.** Patient characteristics and procedural data before and after implementation of the revised platelet transfusion threshold in procedures performed between 2020 and 2026. Values are number (proportion), mean (SD) or median (IQR [range]).

|  | **Overall, n = 615** | **Before new policy, n = 338** | **After new policy,**  **n = 277** | **p value** |
| --- | --- | --- | --- | --- |
| Age; y | 57 (16) | 58 (16) | 56 (16) | 0.169 |
| BMI; kg/m^2^ | 26 (4.8) | 26 (4.6) | 27 (4.9) | 0.046 |
| Missing | 33 (5.4%) | 23 (6.8%) | 10 (3.6%) | 0.080 |
| Gender; female | 269 (44%) | 135 (40%) | 134 (48%) | 0.036 |
| **Diagnosis** |  |  |  |  |
| AML | 214 (35%) | 111 (33%) | 103 (37%) | 0.260 |
| ALL | 56 (9.1%) | 26 (7.7%) | 30 (11%) | 0.178 |
| CML | 18 (2.9%) | 11 (3.3%) | 7 (2.5%) | 0.594 |
| CLL | 14 (2.3%) | 11 (3.3%) | 3 (1.1%) | 0.072 |
| Lymphoma | 86 (14%) | 54 (16%) | 32 (12%) | 0.116 |
| MDS | 36 (5.9%) | 16 (4.7%) | 20 (7.2%) | 0.191 |
| MPN | 7 (1.1%) | 6 (1.8%) | 1 (0.4%) | 0.136 |
| Multiple myeloma | 123 (20%) | 67 (20%) | 56 (20%) | 0.903 |
| Other^†^ | 61 (9.9%) | 36 (11%) | 25 (9.0%) | 0.502 |
| **Vein for insertion** |  |  |  |  |
| Subclavian/axillary | 449 (75%) | 229 (70%) | 220 (81%) | 0.002 |
| Internal jugular | 152 (25%) | 99 (30%) | 53 (19%) | 0.003 |
| External jugular | 1 (0.2%) | 1 (0.3%) | 0 (0.0%) | 1.000 |
| Femoral | 0 (0.0%) | 0 (0.0%) | 0 (0.0%) | n/a |
| Missing | 13 (2.1%) | 9 (2.7%) | 4 (1.4%) | 0.296 |
| Side of insertion; right | 497 (83%) | 263 (80%) | 234 (86%) | 0.063 |
| Missing | 13 (2.1%) | 9 (2.7%) | 4 (1.4%) | 0.296 |
| **Catheter type** |  |  |  |  |
| 1 lumen | 93 (15%) | 87 (26%) | 6 (2.2%) | <0.001 |
| 2 lumen | 470 (78%) | 217 (66%) | 253 (93%) | <0.001 |
| 3 lumen | 8 (1.3%) | 1 (0.3%) | 7 (2.6%) | 0.026 |
| 5 lumen | 2 (0.3%) | 2 (0.6%) | 0 (0.0%) | 0.504 |
| Dialysis | 29 (4.8%) | 23 (7%) | 6 (2.2%) | 0.007 |
| Missing | 13 (2.1%) | 8 (2.4%) | 5 (1.8%) | 0.630 |
| **Needle passes** |  |  |  |  |
| 1 | 446 (76%) | 234 (74%) | 212 (78%) | 0.213 |
| 2 | 91 (15%) | 57 (18%) | 34 (13%) | 0.069 |
| 3 | 31 (5.3%) | 16 (5.0%) | 15 (5.5%) | 0.792 |
| ≥4 | 20 (3.4%) | 10 (3.2%) | 10 (3.7%) | 0.721 |
| Missing | 27 (4.4%) | 21 (6.2%) | 6 (2.2%) | 0.015 |
| **Insertion method** |  |  |  |  |
| Real-time ultrasound | 597 (99%) | 326 (99%) | 271 (99%) | 1.000 |
| Other^‡^ | 5 (0.8%) | 3 (0.9%) | 2 (0.7%) | 1.000 |
| Missing | 13 (2.1%) | 9 (2.7%) | 4 (1.4%) | 0.296 |
| Operator experience >5 years | 478 (79%) | 274 (83%) | 204 (75%) | 0.015 |
| Missing | 11 (1.8%) | 7 (2.1%) | 4 (1.4%) | 0.762 |
| Treated with anticoagulants^§^ | 55 (8.9%) | 35 (10%) | 20 (7.2%) | 0.175 |
| Prophylactic tranexamic acid | 6 (1.0%) | 5 (1.5%) | 1 (0.4%) | 0.230 |
| Haemoglobin, g/l | 93 (83–110 [38–150]) | 93 (84–110 [49–150]) | 93 (82–110 [38–150]) | 0.451 |
| Missing | 72 (12%) | 48 (14%) | 24 (8.7%) | 0.034 |
| INR | 1.1 (1–1.2 [0.8–2.4]) | 1.1 (1–1.2 [0.8–2.4]) | 1.1 (1–1.2 [0.8–2.3]) | 0.029 |
| Missing | 108 (18%) | 77 (23%) | 31 (11%) | <0.001 |
| aPTT; s | 26 (24–28 [20–66]) | 26 (24–28 [20–46]) | 26 (24–28 [20–66]) | 0.143 |
| Missing | 134 (22%) | 84 (25%) | 50 (18%) | 0.042 |
| Fibrinogen, g/l | 3.5 (2.6–4.4 [0.3–9]) | 3.6 (2.7–4.3 [0.5–7.8]) | 3.5 (2.4–4.4 [0.3–9]) | 0.733 |
| Missing | 239 (39%) | 144 (43%) | 95 (34%) | 0.035 |
| Preprocedural PLC; × 10⁹/l | 92 (37–200 [5–820]) | 87 (38–210 [5–820]) | 96 (38–200 [5–730]) | 0.906 |
| Missing | 49 (8%) | 35 (10%) | 14 (5.1%) | 0.016 |
| Preprocedural PLC <10 × 10⁹/l | 21 (3.7%) | 13 (4.3%) | 8 (3.0%) | 0.433 |
| Preprocedural PLC 10–50 × 10⁹/l | 169 (30%) | 87 (29%) | 82 (31%) | 0.523 |
| Preprocedural PLC >50 × 10⁹/l | 376 (66%) | 203 (67%) | 173 (66%) | 0.760 |
| aPTT >43 s | 7 (1.5%) | 1 (0.4%) | 6 (2.6%) | 0.056 |
| INR >1.8 | 7 (1.4%) | 3 (1.1%) | 4 (1.6%) | 0.717 |

Abbreviations: AML, acute myeloid leukaemia; ALL, acute lymphoblastic leukaemia; CML, chronic myeloid leukaemia; CLL, chronic lymphocytic leukaemia; MDS, myelodysplastic syndrome; MPN, myeloproliferative neoplasm; INR, international normalised ratio; aPTT, activated partial thromboplastin time; PLC, platelet count.

^†^ Seminoma and non-seminoma, sickle cell anaemia, Erdheim–Chester disease, aplastic anaemia, multiple sclerosis, hemophagocytic lymphohistiocytosis, myelofibrosis, mixed-phenotype acute leukaemia, acquired haemophilia, and plasmacytoma.

^‡^ Changes over wire, anatomic landmark technique, and ultrasound guidance only before insertion.

^§^ Platelet inhibitors, warfarin, non-vitamin K antagonist oral anticoagulants within 3 days before CVC insertion, or low-molecular-weight heparin (therapeutic doses within 24 h or prophylactic doses within 12 h before the procedure).

**Table S2.** Bleeding, transfusion and mechanical complication outcomes before and after implementation of the revised platelet transfusion threshold in procedures performed between 2020 and 2026. Values are number (proportion), number/total (proportion) or median (IQR [range]).

|  | **Overall**  **n = 615** | **Before new policy,**  **n = 338** | **After new policy,**  **n = 277** | **ARD or median difference** | **95%CI** | **p value** |
| --- | --- | --- | --- | --- | --- | --- |
| **Primary outcomes** |  |  |  |  |  |  |
| Grade 3 bleeding event; n (% (95%CI)) ^†^ | 5 (0.8% (0.3–1.9)) | 5 (1.5% (0.5–3.4)) | 0 (0.0% (0.0–1.3)) | -1.5 | -3.4 to 0.1 | 0.068 |
| Grade 4 bleeding event; n (% (95%CI)) ^†^ | 0 (0.0% (0.0–0.6)) | 0 (0.0% (0.0–1.1)) | 0 (0.0% (0.0–1.3)) | 0.0 | -1.1 to 1.4 | 1.000 |
| Grade 3–4 bleeding event; n (% (95%CI)) ^†^ | 5 (0.8% (0.3–1.9)) | 5 (1.5% (0.5–3.4)) | 0 (0.0% (0.0–1.3)) | -1.5 | -3.4 to 0.1 | 0.068 |
| **Secondary outcomes** |  |  |  |  |  |  |
| Grade 2 bleeding event | 42 (6.8%) | 24 (7.1%) | 18 (6.5%) | -0.6 | -4.6 to 3.6 | 0.768 |
| Preprocedural PLC (transfused insertions);  × 10⁹/l ^‡^ | 102; 21 (13–39 [5–110]) | 84; 23 (15–41 [5–110]) | 18; 16 (10–21 [5–56]) | 8.0 | 0.0 to 16.0 | 0.036 |
| **CVC insertions within each PLC interval receiving preprocedural platelet transfusion** |  |  |  |  |  |  |
| All | 102 (17%) | 84 (25%) | 18 (6.5%) | -18.4 | -23.8 to -12.8 | <0.001 |
| <10 × 10⁹/l | 16/21 (76%) | 11/13 (85%) | 5/8 (62%) | -22.1 | -55.9 to 13.8 | 0.325 |
| 10–50 × 10⁹/l | 75/169 (44%) | 63/87 (72%) | 12/82 (15%) | -57.8 | -68.0 to -44.0 | <0.001 |
| >50 × 10⁹/l | 11/376 (2.9%) | 10/203 (4.9%) | 1/173 (0.6%) | -4.3 | -8.3 to -0.9 | 0.013 |
| Platelet transfusion within 24 h after CVC | 58 (9.4%) | 24 (7.1%) | 34 (12%) | 5.2 | 0.5 to 10.1 | 0.029 |
| **Preprocedural units of platelet transfusions** |  |  |  |  |  |  |
| No transfusion | 513 (83%) | 254 (75%) | 259 (94%) | 18.4 | 12.8 to 23.8 | <0.001 |
| One unit | 93 (15%) | 78 (23%) | 15 (5.4%) | -17.7 | -22.9 to -12.3 | <0.001 |
| Two units | 9 (1.5%) | 6 (1.8%) | 3 (1.1%) | -0.7 | -2.9 to 1.6 | 0.524 |
| Total units | 111 | 90 | 21 | n/a | n/a | n/a |
| Per patient | 0.18 | 0.27 | 0.08 | n/a | n/a | n/a |
| **Other mechanical complications** |  |  |  |  |  |  |
| Pneumothorax | 4 (0.7%) | 2 (0.6%) | 2 (0.7%) | 0.1 | -1.5 to 2.1 | 1.000 |
| Arterial puncture | 6 (1.0%) | 5 (1.5%) | 1 (0.4%) | -1.1 | -3.1 to 0.7 | 0.230 |
| Non-persistent nerve injury | 2 (0.3%) | 1 (0.3%) | 1 (0.4%) | 0.1 | -1.3 to 1.7 | 1.000 |
| Arrhythmias requiring intervention | 0 (0.0%) | 0 (0.0%) | 0 (0.0%) | 0.0 | -1.1 to 1.4 | 1.000 |

Abbreviations: ARD, absolute risk difference; PLC, platelet count; CVC, central venous catheter; n/a, not applicable.

^†^ Clopper–Pearson 95% CI are presented for proportions with sparse data or zero events.
^‡^ Median difference presented as Hodges–Lehmann estimate with 95% CI.

**Table S3.** Bleeding events before and after implementation of the revised platelet transfusion threshold in central venous catheter insertions with preprocedural platelet counts of 10–50 × 10⁹/l. Values are number (proportion).

|  | **Overall,**  **n = 297** | **Before new policy,**  **n = 215** | **After new policy,**  **n = 82** | **ARD** | **95%CI** | **p value** |
| --- | --- | --- | --- | --- | --- | --- |
| Grade 2 bleeding event | 36 (12%) | 28 (13%) | 8 (9.8%) | -3.3 | -10.3 to 5.9 | 0.441 |
| Grade 3–4 bleeding events | 10 (3.4%) | 10 (4.7%) | 0 (0.0%) | -4.7 | -8.3 to 0.3 | 0.067 |
| Grade 2–4 bleeding events | 46 (15%) | 38 (18%) | 8 (9.8%) | -7.9 | -15.3 to 1.6 | 0.092 |

Abbreviations: ARD, absolute risk difference.

**Table S4.** Platelet transfusions administered within 24 h after central venous catheter insertion according to preprocedural platelet count before and after implementation of the revised platelet transfusion threshold in procedures performed between 2020 and 2026. Values are number (proportion) or number/total (proportion).

|  | **Overall**  **N = 58** | **Before**  **N = 24** | **After**  **N = 34** | **p value** |
| --- | --- | --- | --- | --- |
| <10 × 10⁹/l | 9 (16%) | 4 (17%) | 5 (15%) | 1.000 |
| Grade 3–4 bleeding event | 0/9 (0.0%) | 0/4 (0.0%) | 0/5 (0.0%) | n/a |
| Grade 2 bleeding event | 4/9 (44%) | 1/4 (25%) | 3/5 (60%) | 0.524 |
| 10–50 × 10⁹/l | 41 (71%) | 15 (62%) | 26 (76%) | 0.250 |
| Grade 3–4 bleeding event | 1/41 (2.4%) | 1/15 (6.7%) | 0/26 (0.0%) | 0.366 |
| Grade 2 bleeding event | 5/41 (12%) | 2/15 (13%) | 3/26 (12%) | 1.000 |
| >50 × 10⁹/l | 7 (12%) | 4 (17%) | 3 (8.8%) | 0.432 |
| Grade 3–4 bleeding event | 2/7 (29%) | 2/4 (50%) | 0/3 (0.0%) | 0.429 |
| Grade 2 bleeding event | 1/7 (14%) | 1/4 (25%) | 0/3 (0.0%) | 1.000 |
| Missing | 1 (1.7%) | 1 (4.2%) | 0 (0.0%) | 0.414 |

**Table S5.** Patient characteristics and procedural data before and after implementation of the revised platelet transfusion threshold, including all catheter insertions. Values are number (proportion), mean (SD) or median (IQR [range]).

|  | **Overall, n = 1315** | **Before new policy, n = 927** | **After new policy,**  **n = 388** | **p value** |
| --- | --- | --- | --- | --- |
| Age; y | 55 (16) | 55 (16) | 54 (16) | 0.257 |
| BMI; kg/m^2^ | 26 (5.0) | 26 (4.9) | 27 (5.3) | 0.072 |
| Missing | 45 (3.4%) | 33 (3.6%) | 12 (3.1%) | 0.671 |
| Gender; female | 557 (42%) | 383 (41%) | 174 (45%) | 0.238 |
| **Diagnosis** |  |  |  |  |
| AML | 549 (42%) | 387 (42%) | 162 (42%) | 0.999 |
| ALL | 165 (13%) | 115 (12%) | 50 (13%) | 0.810 |
| CML | 41 (3.1%) | 31 (3.3%) | 10 (2.6%) | 0.466 |
| CLL | 34 (2.6%) | 30 (3.2%) | 4 (1.0%) | 0.022 |
| Lymphoma | 145 (11%) | 100 (11%) | 45 (12%) | 0.669 |
| MDS | 54 (4.1%) | 34 (3.7%) | 20 (5.2%) | 0.215 |
| MPN | 10 (0.8%) | 8 (0.9%) | 2 (0.5%) | 0.732 |
| Multiple myeloma | 168 (13%) | 105 (11%) | 63 (16%) | 0.015 |
| Other^†^ | 149 (11%) | 117 (13%) | 32 (8.2%) | 0.022 |
| **Vein for insertion** |  |  |  |  |
| Subclavian/axillary | 995 (77%) | 696 (76%) | 299 (78%) | 0.485 |
| Internal jugular | 300 (23%) | 217 (24%) | 83 (22%) | 0.412 |
| External jugular | 3 (0.2%) | 1 (0.1%) | 2 (0.5%) | 0.210 |
| Femoral | 1 (0.1%) | 1 (0.1%) | 0 (0.0%) | 1.000 |
| Missing | 16 (1.2%) | 12 (1.3%) | 4 (1.0%) | 0.790 |
| Side of insertion; right | 817 (63%) | 517 (57%) | 300 (78%) | <0.001 |
| Missing | 17 (1.3%) | 13 (1.4%) | 4 (1.0%) | 0.587 |
| **Catheter type** |  |  |  |  |
| 1 lumen | 537 (42%) | 525 (58%) | 12 (3.1%) | <0.001 |
| 2 lumen | 648 (51%) | 298 (33%) | 350 (91%) | <0.001 |
| 3 lumen | 21 (1.6%) | 13 (1.4%) | 8 (2.1%) | 0.408 |
| 5 lumen | 17 (1.3%) | 13 (1.4%) | 4 (1.0%) | 0.564 |
| Dialysis | 58 (4.5%) | 49 (5.5%) | 9 (2.3%) | 0.014 |
| Missing | 34 (2.6%) | 29 (3.1%) | 5 (1.3%) | 0.055 |
| **Needle passes** |  |  |  |  |
| 1 | 907 (73%) | 620 (72%) | 287 (76%) | 0.143 |
| 2 | 198 (16%) | 148 (17%) | 50 (13%) | 0.081 |
| 3 | 84 (6.8%) | 57 (6.6%) | 27 (7.1%) | 0.732 |
| ≥4 | 51 (4.1%) | 37 (4.3%) | 14 (3.7%) | 0.631 |
| Missing | 75 (5.7%) | 65 (7.0%) | 10 (2.6%) | 0.002 |
| **Insertion method** |  |  |  |  |
| Real-time ultrasound | 1130 (87%) | 751 (82%) | 379 (99%) | <0.001 |
| Other^‡^ | 168 (13%) | 163 (18%) | 5 (1.3%) | <0.001 |
| Missing | 17 (1.3%) | 13 (1.4%) | 4 (1.0%) | 0.587 |
| Operator experience >5 years | 936 (73%) | 646 (73%) | 290 (76%) | 0.263 |
| Missing | 40 (3.0%) | 36 (3.9%) | 4 (1.0%) | 0.006 |
| Treated with anticoagulants^§^ | 99 (7.5%) | 72 (7.8%) | 27 (7.0%) | 0.612 |
| Prophylactic tranexamic acid | 10 (1.2%) | 9 (2.2%) | 1 (0.26%) | 0.021 |
| Haemoglobin, g/l | 96 (85–110 [38–150]) | 97 (86–110 [49–150]) | 94 (83–110 [38–150]) | 0.210 |
| Missing | 163 (12%) | 132 (14%) | 31 (8.0%) | 0.002 |
| INR | 1.1 (1.0–1.2 [0.8–3.8]) | 1.1 (1.0–1.2 [0.8–3.8]) | 1.1 (1.0–1.2 [0.8–2.3]) | 0.078 |
| Missing | 222 (17%) | 162 (17%) | 60 (15%) | 0.374 |
| aPTT; s | 26 (24–29 [20–67]) | 26 (24–29 [20–67]) | 26 (24–28 [20–66]) | 0.955 |
| Missing | 264 (20%) | 168 (18%) | 96 (25%) | 0.006 |
| Fibrinogen, g/l | 3.6 (2.6–4.4 [0.3–9.0]) | 3.5 (2.6–4.4 [0.3–9.0]) | 3.6 (2.6–4.5 [0.3–9.0]) | 0.307 |
| Missing | 616 (47%) | 467 (50%) | 149 (38%) | <0.001 |
| Preprocedural PLC; × 10⁹/l | 88 (33–190 [5–870]) | 84 (33–190 [5–870]) | 98 (32–190 [5–730]) | 0.779 |
| Missing | 73 (5.6%) | 53 (5.7%) | 20 (5.2%) | 0.684 |
| Preprocedural PLC <10 × 10⁹/l | 53 (4.3%) | 38 (4.3%) | 15 (4.1%) | 0.829 |
| Preprocedural PLC 10–50 × 10⁹/l | 391 (31%) | 272 (31%) | 119 (32%) | 0.674 |
| Preprocedural PLC >50 × 10⁹/l | 798 (64%) | 564 (65%) | 234 (64%) | 0.751 |
| aPTT >43 s | 15 (1.4%) | 8 (1.1%) | 7 (2.4%) | 0.142 |
| INR >1.8 | 12 (1.1%) | 8 (1.0%) | 4 (1.2%) | 0.759 |

Abbreviations: AML, acute myeloid leukaemia; ALL, acute lymphoblastic leukaemia; CML, chronic myeloid leukaemia; CLL, chronic lymphocytic leukaemia; MDS, myelodysplastic syndrome; MPN, myeloproliferative neoplasm; INR, international normalised ratio; aPTT, activated partial thromboplastin time; PLC, platelet count.

^†^ Seminoma and non-seminoma, sickle cell anaemia, Erdheim–Chester disease, aplastic anaemia, multiple sclerosis, hemophagocytic lymphohistiocytosis, myelofibrosis, mixed-phenotype acute leukaemia, acquired haemophilia, and plasmacytoma.

^‡^ Changes over wire, anatomic landmark technique, and ultrasound guidance only before insertion.

^§^ Platelet inhibitors, warfarin, non-vitamin K antagonist oral anticoagulants within 3 days before CVC insertion, or low-molecular-weight heparin (therapeutic doses within 24 h or prophylactic doses within 12 h before the procedure)

**Table S6.** Bleeding, transfusion and mechanical complication outcomes before and after implementation of the revised platelet transfusion threshold, including all catheter insertions. Values are number (proportion), number/total (proportion) or median (IQR [range]).

|  | **Overall**  **n = 1315** | **Before new policy,**  **n = 927** | **After new policy,**  **n = 388** | **ARD or median difference** | **95% CI** | **p value** |
| --- | --- | --- | --- | --- | --- | --- |
| **Primary outcomes** |  |  |  |  |  |  |
| Grade 3 bleeding event; n (% (95% CI)) ^†^ | 11 (0.8% (0.4–1.5)) | 11 (1.2% (0.6–2.1)) | 0 (0.0% (0.0–0.9)) | -1.2 | -2.1 to -0.1 | 0.040 |
| Grade 4 bleeding event; n (% (95% CI)) ^†^ | 3 (0.2% (0.0–0.7)) | 3 (0.3% (0.1–0.9)) | 0 (0.0% (0.0–0.9)) | -0.3 | -0.9 to 0.7 | 0.560 |
| Grade 3–4 bleeding event; n (% (95% CI)) ^†^ | 14 (1.1% (0.6–1.8)) | 14 (1.5% (0.8–2.5)) | 0 (0.0% (0.0–0.9)) | -1.5 | -2.5 to -0.4 | 0.014 |
| **Secondary outcomes** |  |  |  |  |  |  |
| Grade 2 bleeding event | 105 (8.0%) | 76 (8.2%) | 29 (7.5%) | -0.7 | -3.7 to 2.7 | 0.659 |
| Preprocedural PLC (transfused insertions);  × 10⁹/l ^‡^ | 22 (13–38 [5–299]) | 23 (15–39 [5–226]) | 16 (9–26 [5–299]) | 6.0 | 1.0 to 12.0 | 0.026 |
| **CVC insertions within each PLC interval receiving preprocedural platelet transfusion** |  |  |  |  |  |  |
| All | 298 (23%) | 268 (29%) | 30 (7.7%) | -21.2 | -24.9 to -17.0 | <0.001 |
| <10 × 10⁹/l | 42/53 (79%) | 34/38 (89%) | 8/15 (53%) | -36.1 | -60.2 to -10.4 | 0.007 |
| 10–50 × 10⁹/l | 228/391 (58%) | 208/272 (76%) | 20/119 (17%) | -59.7 | -67.0 to -50.2 | <0.001 |
| >50 × 10⁹/l | 28/798 (3.5%) | 26/564 (4.6%) | 2/234 (0.9%) | -3.8 | -5.9 to -1.1 | 0.006 |
| Platelet transfusion within 24 h after CVC | 168 (13%) | 121 (13%) | 47 (12%) | -0.9 | -4.6 to 3.2 | 0.642 |
| **Preprocedural units of platelet transfusions** |  |  |  |  |  |  |
| No transfusion | 1017 (77%) | 659 (71%) | 358 (92%) | 21.2 | 17.0 to 24.9 | <0.001 |
| One unit | 277 (21%) | 251 (27%) | 26 (6.7%) | -20.4 | -24.0 to -16.3 | <0.001 |
| Two units | 21 (1.6%) | 17 (1.8%) | 4 (1.0%) | -0.8 | -2.1 to 0.9 | 0.344 |
| Total units | 319 | 285 | 34 | n/a | n/a | n/a |
| Per patient | 0.24 | 0.31 | 0.09 | n/a | n/a | n/a |
| **Other mechanical complications** |  |  |  |  |  |  |
| Pneumothorax | 7 (0.5%) | 4 (0.4%) | 3 (0.8%) | 0.3 | -0.5 to 1.8 | 0.427 |
| Arterial puncture | 26 (2.0%) | 24 (2.6%) | 2 (0.5%) | -2.1 | -3.4 to -0.5 | 0.015 |
| Non-persistent nerve injury | 6 (0.5%) | 4 (0.4%) | 2 (0.5%) | 0.1 | -0.7 to 1.5 | 1.000 |
| Arrhythmias requiring intervention | 3 (0.2%) | 3 (0.3%) | 0 (0.0%) | -0.3 | -0.9 to 0.7 | 0.560 |

Abbreviations: ARD, absolute risk difference; PLC, platelet count; CVC, central venous catheter; n/a, not applicable.

^†^ Clopper–Pearson 95% CI are presented for proportions with sparse data or zero events.
^‡^ Median difference presented as Hodges–Lehmann estimate with 95% CI.

**Table S7.** Patient characteristics and procedural data for patients with grade 3–4 bleeding events. Values are number (proportion), mean (SD) or median (IQR [range]).

|  | **Grade 3–4 bleeding events n = 14** | **No grade 2–4 bleeding events n = 899** |
| --- | --- | --- |
| Age; y | 57 (18) | 56 (16) |
| BMI; kg/m^2^ | 25 (4.3) | 26 (4.9) |
| Gender; female | 5 (36%) | 390 (43%) |
| **Diagnosis** |  |  |
| AML | 8 (57%) | 343 (38%) |
| ALL | 3 (21%) | 96 (11%) |
| MDS | 2 (14%) | 45 (5.0%) |
| Other^†^ | 1 (7.1%) | 107 (12%) |
| **Vein for insertion** |  |  |
| Subclavian/axillary | 11 (79%) | 710 (80%) |
| Internal jugular | 3 (21%) | 173 (20%) |
| Side of insertion; right | 7 (50%) | 592 (67%) |
| **Catheter type** |  |  |
| 1 lumen | 10 (71%) | 363 (41%) |
| 2 lumen | 4 (29%) | 458 (52%) |
| **Insertion method** |  |  |
| Real-time ultrasound | 11 (79%) | 779 (88%) |
| Other^†^ | 3 (21%) | 106 (12%) |
| Operator experience >5 years | 13 (93%) | 633 (73%) |
| Preprocedural PLC; × 10⁹/l | 27 (16–49 [11–85]) | 92 (39–200 [5–820]) |
| Preprocedural platelet transfusion | 10 (71%) | 188 (21%) |

Abbreviations: AML, acute myeloid leukaemia; ALL, acute lymphoblastic leukaemia; MDS, myelodysplastic syndrome; PLC, platelet count.

^†^ Seminoma and non-seminoma, sickle cell anaemia, Erdheim–Chester disease, aplastic anaemia, multiple sclerosis, hemophagocytic lymphohistiocytosis, myelofibrosis, mixed-phenotype acute leukaemia, acquired haemophilia, and plasmacytoma.
